# The Role of Angiotensin II and Cyclic AMP in Alveolar Active Sodium Transport

**DOI:** 10.1371/journal.pone.0134175

**Published:** 2015-07-31

**Authors:** Reem Ismael-Badarneh, Julia Guetta, Geula Klorin, Gidon Berger, Niroz Abu-saleh, Zaid Abassi, Zaher S. Azzam

**Affiliations:** 1 Internal Medicine "B", Rambam: Human Health Care Campus, Haifa, Israel; 2 Department of Physiology, Ruth & Bruce Rappaport Faculty of Medicine, Technion, Israel Institute of Technology, Haifa, Israel; 3 The Rappaport Family Institute for Research in the Medical Sciences, Technion, Israel Institute of Technology, Haifa, Israel; Max-Delbrück Center for Molecular Medicine (MDC), GERMANY

## Abstract

Active alveolar fluid clearance is important in keeping airspaces free of edema. Angiotensin II plays a role in the pathogenesis of hypertension, heart failure and others. However, little is known about its contribution to alveolar fluid clearance. Angiotensin II effects are mediated by two specific receptors; AT_1_ and AT_2_. The localization of these two receptors in the lung, specifically in alveolar epithelial cells type II, was recently reported. We hypothesize that Angiotensin II may have a role in the regulation of alveolar fluid clearance. We investigated the effect of Angiotensin II on alveolar fluid clearance in rats using the isolated perfused lung model and isolated rat alveolar epithelial cells. The rate of alveolar fluid clearance in control rats was 8.6% ± 0.1 clearance of the initial volume and decreased by 22.5%, 28.6%, 41.6%, 48.7% and 39% in rats treated with 10^-10^ M, 10^-9^ M, 10^-8^ M, 10^-7^ M or 10^-6^ M of Ang II respectively (P < 0.003). The inhibitory effect of Angiotensin II was restored in losartan, an AT_1_ specific antagonist, pretreated rats, indicating an AT_1_ mediated effect of Ang II on alveolar fluid clearance. The expression of Na,K-ATPase proteins and cAMP levels in alveolar epithelial cells were down-regulated following the administration of Angiotensin II; suggesting that cAMP may be involved in AngII-induced reduced Na,K-ATPase expression, though the contribution of additional factors could not be excluded. We herein suggest a novel mechanism of clinical relevance by which angiotensin adversely impairs the ability of the lungs to clear edema.

## Introduction

Pulmonary edema is a life threatening condition of various major etiologies, such as, congestive heart failure (CHF), which has a high morbidity and mortality rates, especially among the aging population [1]. Understanding the basic mechanisms leading to the development of CHF and its complications, including pulmonary edema, is therefore crucial for optimizing medical treatment of CHF and for designing novel therapies in an attempt to improve the outcome of the disease. Pulmonary edema is the condition of excess fluids accumulated in alveolar spaces, derived by changes in the hydrostatic and/or oncotic pressure gradients across the pulmonary circulation and the lung interstitium [2, 3], by increased lung permeability, or disturbed resolution due to decreased alveolar fluid clearance (AFC) [4]. Active AFC is important in keeping the airspace free of edema. It is mediated via active sodium transport, a process by which Na^+^ is extruded out of the alveolar airspace by epithelial transport proteins, including apical epithelial Na^+^ channels (ENaC) and basolateral Na,K-ATPases with water following to restore isosmotic conditions.

Since the early 1980s, the importance of vasoconstrictor neuro-hormonal systems, including Angiotensin II (Ang II), in the pathogenesis of CHF has been increasingly recognized [5–7]. It is well established that Ang II is increased in heart failure [8]. The contribution of Ang II to the pathogenesis of hypertension, cardiac hypertrophy and heart failure, alongside with Ang II role in body fluids homeostasis [9, 10], and the previously published existence of Ang II receptors in AEC II[11], led us to investigate the role of Ang II on AFC in AEC II, and the molecular mechanisms by which Ang II exerts its effect on AFC.

## Materials and Methods

### Ethics Statement

The experiments were performed on adult male Sprague Dawley rats (275–350g) (Harlan Laboratories Ltd. Jerusalem, Israel). Rats were provided water and food ad libitum. The use of animals in this study was approved by the Technion Institutional Animal Care and Use Committee, and the animals were treated according to NIH guidelines.

In order to avoid sacrificing large amounts of animals; we used to perform an informal interim analysis when each group reached the number of 4 animals. This approach could result in a large reduction in animal use by stopping large studies that are very unlikely to produce positive evidence to support a hypothesis. This approach is in line with the guidelines of the National Centre for the Replacement, Refinement and Reduction of Animals in Research (London, UK).

### Isolated Perfused liquid-filled lung model

An AFC rate and alveolar epithelial permeability were measured by utilizing the Isolated perfused liquid-filled lung model as described elsewhere [12].

### Experimental groups

To investigate the effect of angiotensin II on AFC, we established a dose response curve of Ang II perfused through the pulmonary circulation and instilled to the lungs: 10^−10^, 10^−9^, 10^−8^, 10^−7^ and 10^-6^M. While 10^−9^ M Ouabain (Cat# 1076, Tocris Biosiences) and 10^-6^M Amiloride (Cat# A7410, Sigma Aldrich) were given through the tail vein 30 minutes prior to sacrificing the rat, 10^−6^ M Norepinephrine (Cat# 1468501, Sigma Aldrich) was perfused through the pulmonary circulation of the isolated lungs. Losartan (0.02mg/gr rat/day) was added to drink water for three days prior to sacrificing the rat (Cat# 10006594, Cayman chemical). PD123319 was given through the tail vein 30 minutes prior to sacrificing the rat (100μg/kg rat), and perfused through the pulmonary circulation (10μg/ml) since it has a short half time (22 minutes) (Cat# P186, Sigma Aldrich).

### Alveolar epithelial cells type II (AEC II cell) isolation

Alveolar epithelial cells type II were isolated from the lungs of the various study groups based on a method described by Dobbs et al. [13]. Isolated AEC II were incubated for 28 hours at 37°C, and then treated with/without Ang II for 2 hours.

### Cell Lysate and Western Blot Analysis

Cell lysates were homogenized in buffer, resolved in a 10% SDS-PAGE and analyzed by immunoblotting with specific antibodies (Ab); anti-α1-Na,K-ATPase (1μg/ml, Cat# ab7671, Abcam), anti-β-actin (1:1000, Cat# 4970, Cell Signaling Technologies, inc.), anti-angiotensin receptor type 1 (1:500, Cat# sc-1173, Santa Cruz Biotechnologies, inc), anti-αENaC (1:1000, Cat# sc-21012, Santa Cruz Biotechnologies, inc) and anti-αtubulin (1:3000, Cat# ab126165, Abcam). ECL kit (Thermo Scientific, USA) was used to develop the image.

### Immunofluorescence

Alveolar epithelial cells type II were fixed with 0.1% formaldehyde for 10 min at RT, rinsed with 10% Donkey serum-PBS, incubated over-night with primary Ab (α-Na,K-ATPase, 1:100, Cat# ab7671, Abcam) at 4°C, followed by fluorescence-labeled secondary Ab incubation. Dapi was used for nuclear DNA staining. Zeiss Axio observer inverted motorized fluorescent microscope equipped with Hamamtsu Orca R2 and Zeiss HS cameras was used for microscope imaging.

### Immunohistochemistry

Immunohistochemistry experiments were pursued as previously described [14]. AEC II cells were fixed with 95% ethanol on coverslips. Endogenous peroxidase reactive is quenched in 0.3% hydrogen peroxides in wash buffer solution; immunoperoxides staining was performed using a streptavidin-biotin system kit for specific antibodies: AT_1_ receptor Ab (1:200, Cat# sc-1173, Santa Cruz Biotechnologies, INC).

### Cyclic AMP levels

Determination of intracellular cyclic adenosine monophosphate (cAMP) concentrations, AEC II cells were treated with or without Ang II*10^-7^M for 20 min. Cell lysates were harvested after 0.1% HCl treatment and centrifuged. Supernatants were collected and acetylated following the manufacturer's specifications, and then cAMP levels were determined using an immunoassay (Cyclic AMP EIA kit, Cayman Chemicals Co.). Absorbance was measured at 410nm, with Anthos microplate spectrophotometer Zenyth 200. cAMP concentration (pmol/*μ*g protein) for each sample was determined according to the kit's instructions.

### Statistical Analysis

Data are presented as mean values ± SEM. One way ANOVA was used when multiple comparisons were required followed by a multiple comparison test (Tukey) when the F statistic indicated significance. Differences between groups were determined by using paired Student's t test. Results were considered significant when p < 0.05

## Results

### Effect of Angiotensin-II on Alveolar Fluid Clearance

**Fig 1:**
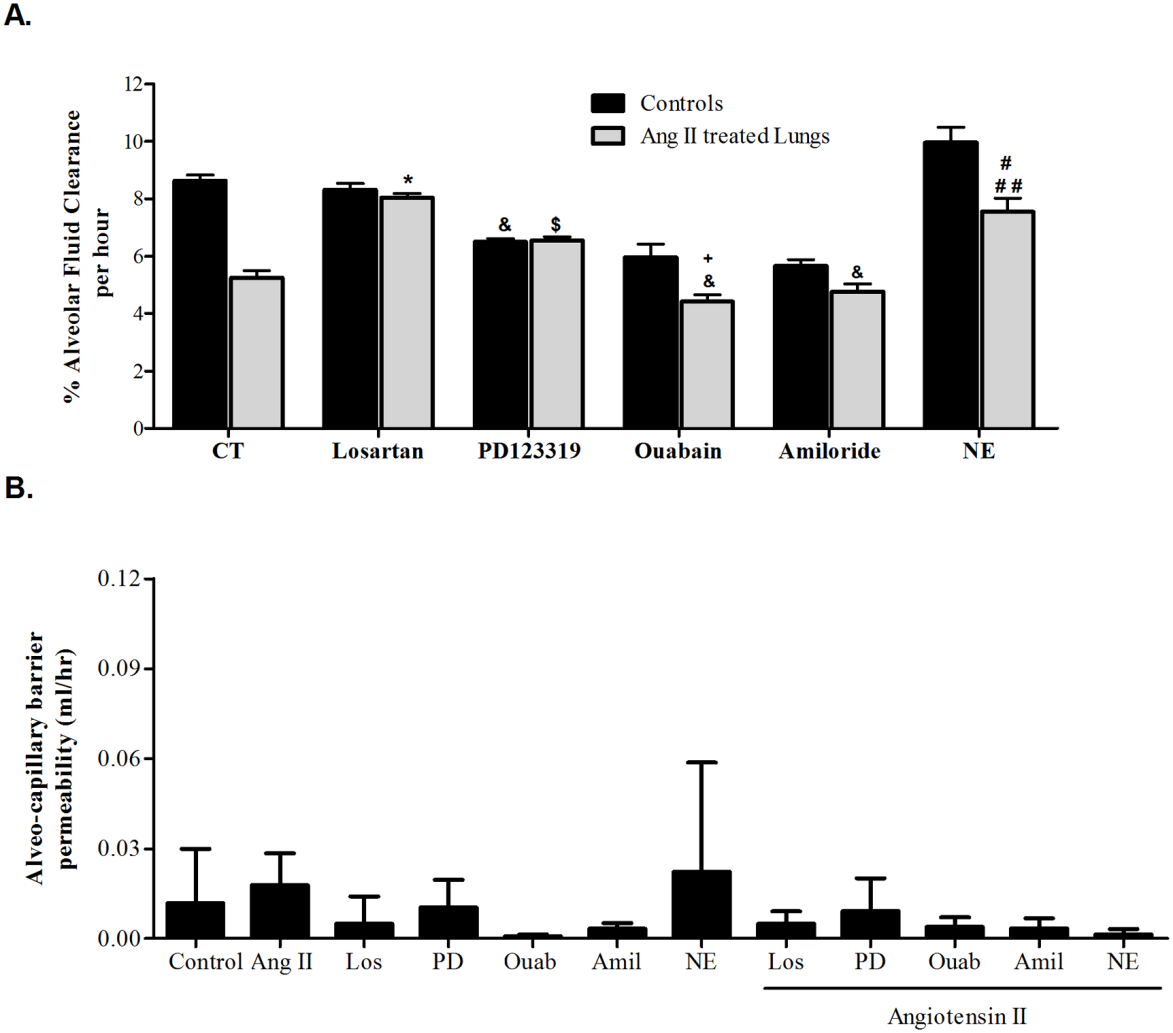
Effect of Ang II on AFC. (A) % Alveolar fluid clearance of the initial instilled volume was decreased in the Ang II groups in a dose dependent manner, from 8.6% ± 0.19 in control rats to 6.66% ± 0.13, 6.15% ± 0.11, 5.03% ± 0.31, 4.42% ± 0.29 and 5.25% ± 0.23 in Ang II (10^−10^ M, 10^-9^M, 10^−8^ M, 10^−7^ M and 10^−6^ M) respectively. * P<0.001 As compared to control group; ** P<0.05 As compared to the rest of 10^−10^ M and 10^−9^ M Ang II treated groups. CT—Control. The bars represent mean ± SEM. (B) The albumin movement across the alveolar-capillary barrier did not differ significantly among the study groups indicating that the barrier was intact. CT—Control. The bars represent mean ± SEM.

As depicted in Fig 1A, we established a dose response curve of Ang II on AFC: 10^−10^ M (n = 4), 10^−9^ M (n = 8), 10^−8^ M (n = 5), 10^−7^ M (n = 4), 10^−6^ M (n = 11).

In Angiotensin II treated lungs, AFC was decreased as compared to control lungs (from 8.6%±0.19 of the initial instilled volume, in control lungs, to 6.66%±0.13, 6.15%±0.11, 5.03%±0.31, 4.42%±0.29 and 5.25%±0.23 (*P*<0.0001) in Ang II 10^−10^ M, 10^−9^ M, 10^−8^ M, 10^−7^ M and 10^−6^ M treated lungs, respectively. There was no statistical difference between the various Ang II treated groups (Fig 1A and S1 Table). The albumin movement across the alveolar-capillary barrier did not differ significantly among the study groups indicating that the barrier was intact (Fig 1B), and that the fluids clearance was driven by the active AFC.

### Angiotensin-II effect is mediated by Angiotensin-II Receptor type 1

To determine whether Ang II signaling is mediated by the AT_1_ receptor, we treated the rats with Losartan (0.02 mg Los/gr rat/day) for three days either alone (n = 5) or in the presence of Ang II (10^-6^M) (n = 5), and then proceeded to measure the rate of AFC.

**Fig 2:**
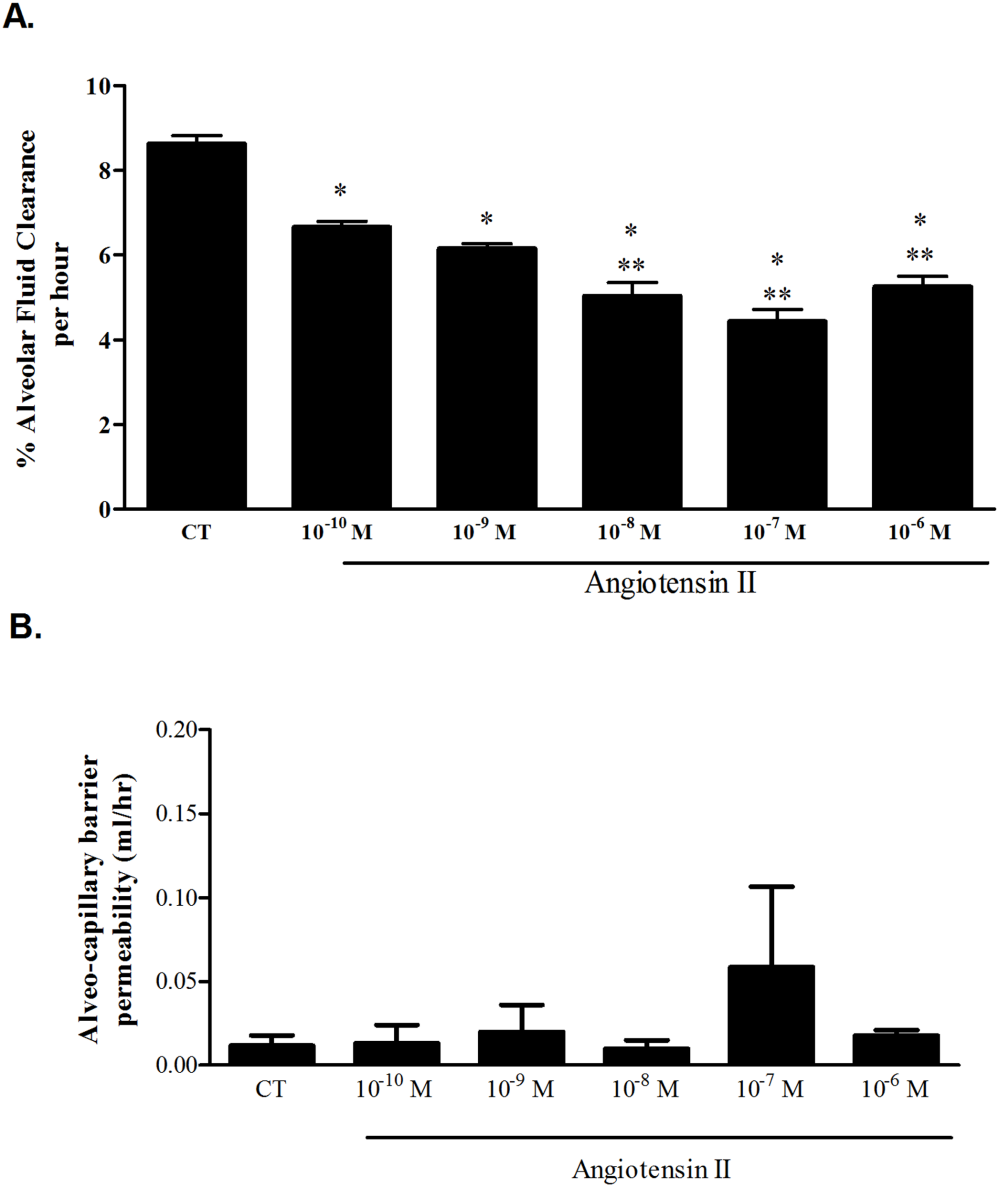
Different interventions effect on AFC. (A) Losartan restored Ang II effect on AFC from 5.25%±0.23 to 8.1%±0.13. AFC was not different in both losartan treated groups. * P<0.001 As compared to control group treated with Ang II. PD123319, AT_2_ receptor antagonist, decreased AFC in both AngII treated (n = 4) and untreated groups (n = 4) (6.54%±0.2 and 6.51%±0.2 respectively). $ P<0.05 as compared to Ang II group, & P<0.001 as compared to control group. Ouabain, the Na,K-ATPase blocker, significantly inhibited AFC in both control and Ang II treated rat lungs (5.9% ± 0.4 and 4.4% ± 0.2 respectively). + P<0.05 as compared to control rat lungs treated with ouabain alone. Amiloride, the sodium channel blocker, significantly reduced AFC in both control and Ang II treated rats as compared to untreated lungs (a 5.6% ± 0.2 and 5.01 ± 0.2 respectively). However, AFC was similar in the two Amiloride treated groups. Activating the adrenergic pathway by norepinephrine 10^-6^M increased the clearance percentage to 14.12% ± 1.8, when compared to control 8.6% ± 0.19. But when Ang II was also added, NE effect was abolished (7.3% ± 0.6). # P<0.05 as compared to control rat lungs treated with norepinephrine alone. ## P<0.0001 as compared to AngII group. CT—Control. Ang II—Angiotensin II. NE—Norepinephrine. The bars represent mean ± SEM. (B) The albumin movement across the alveolar-capillary barrier did not differ significantly among the study groups indicating that the barrier was intact. CT—Control. Ang II—Angiotensin II. Los—Losartan. PD—PD123319. Ouab—Ouabain. Amil—Amiloride. NE—Norepinephrine. The bars represent mean ± SEM.

In rats treated with Losartan, AT_1_ specific antagonist, alone, the rate of AFC was similar to control (8.3% ±0.2 compared to 8.6%±0.19 in control rats) (Fig 2A and S2 Table). But when adding losartan to Ang II (10^-6^M) treated lungs, the inhibitory effect was blocked; AFC was increased from 5.25%±0.23 in Ang II treated lungs to 8.1%±0.13 in rats treated with losartan prior to Ang II administration, P<0.001. The alveolar-capillary barrier was intact; there was no leakage (Fig 2B).

**Fig 3:**
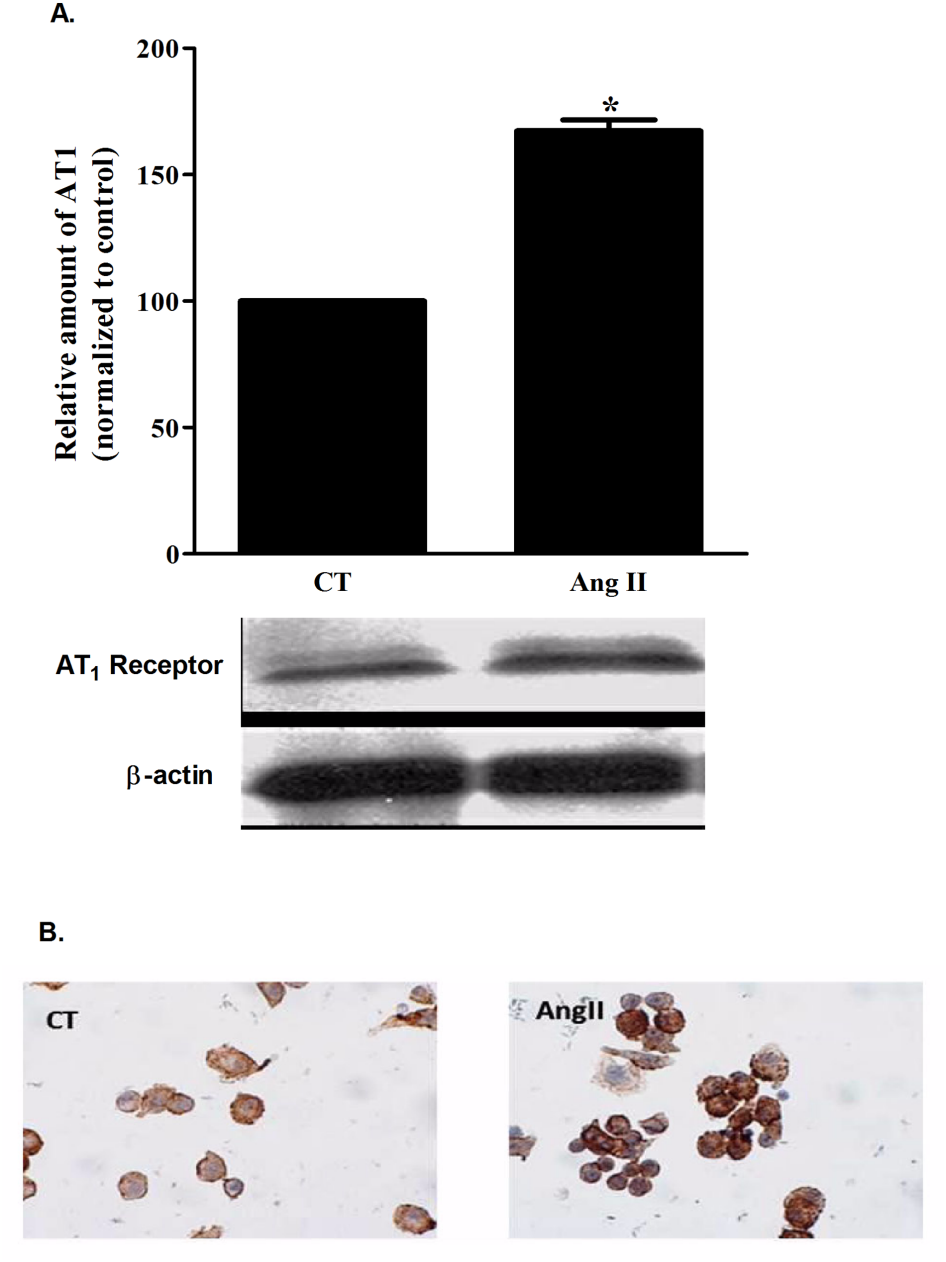
AT_1_ levels. (A) A semi-quantative measure of AT1 protein levels in AEC II by western blotting; AT1 levels were significantly increased by 1.67 fold after Ang II administration. *P<0.0001 as compared to control group. CT—Control. Ang II—Angiotensin II. AT1—Angiotensin-II receptor type 1. The bars represent mean ± SEM. (B) An immunohestochemical staining of AT1 receptor in AEC II cells treated or untreated with Ang II. The representative figure showing stronger staining of AT1 in the Ang II treated group compared to the control. CT—Control. Ang II—Angiotensin II.

This set of experiments provided physiologic evidence that AT_1_ receptors maybe present; therefore we sought those receptors using western blotting and immunofluorescence techniques. As shown in Fig 3B, AT_1_ receptors were detected in AEC II cells; while the addition of Ang II to AEC II cells increased the level of AT_1_ receptor by 1.67 fold (Fig 3A), p<0.001. This Ang II stimulatory effect may be due either to AT_1_ degradation inhibition or to enhanced trafficking of intracellular AT_1_ to the cell surface.

### Effect of Amiloride and Ouabain on Ang II treated rat lungs

To further understand the effect of Ang II on AFC, we investigated the role of the apical Na^+^ channels and the basolateral Na,K-ATPase on Ang II (Fig 2A and S2 Table). Rats treated with Ouabain (10^-9^M) alone (n = 4), showed decreased AFC levels, 5.95% ±0.45 (Fig 2A), but when treated with both Ouabain and Ang II (10^-6^M) (n = 5), AFC levels were further impaired, (4.41%±0.23) (P<0.05). AFC levels in rats treated with 10^-6^M Amiloride alone (n = 4) were also decreased to 5.66%±0.21 (P<0.001 as compared to control group), and to 4.76%±0.27 when treated with both Amiloride and Ang II (n = 6); notably, AFC in the two treated Amiloride groups was not statistically different.

### The interaction between the adrenergic pathways and angiotensin II

To determine whether the adrenergic pathways have an effect on Ang II treated rat lungs; we treated rat lungs with both 10^-6^M norepinephrine (NE) and 10^-6^M Ang II. NE increased the rate of AFC by 1.64 folds (to 14.12%±1.8 of the initial volume) (n = 3), whereas in rat lungs treated with both Ang II and NE (n = 4), the rate AFC was 7.3%±0.6 as compared to 5.25%±0.23 in rat lungs treated with AngII alone; indicating that NE counterbalances Ang II impairment (Fig 2A and S2 Table). Notably, these results are not sufficient to conclude an interaction between both pathways.

The albumin movement across the alveolar-capillary barrier did not differ significantly among the study groups indicating that the barrier was intact (Fig 2B).

### Na,K-ATPase inhibition by Ang II

Since Ang II impaired AFC levels, we investigated its effect on active sodium transport, specifically Na,K-ATPase. AEC II cells were isolated [13], and treated either alone or with Ang II for two hours, then protein levels were measured.

**Fig 4:**
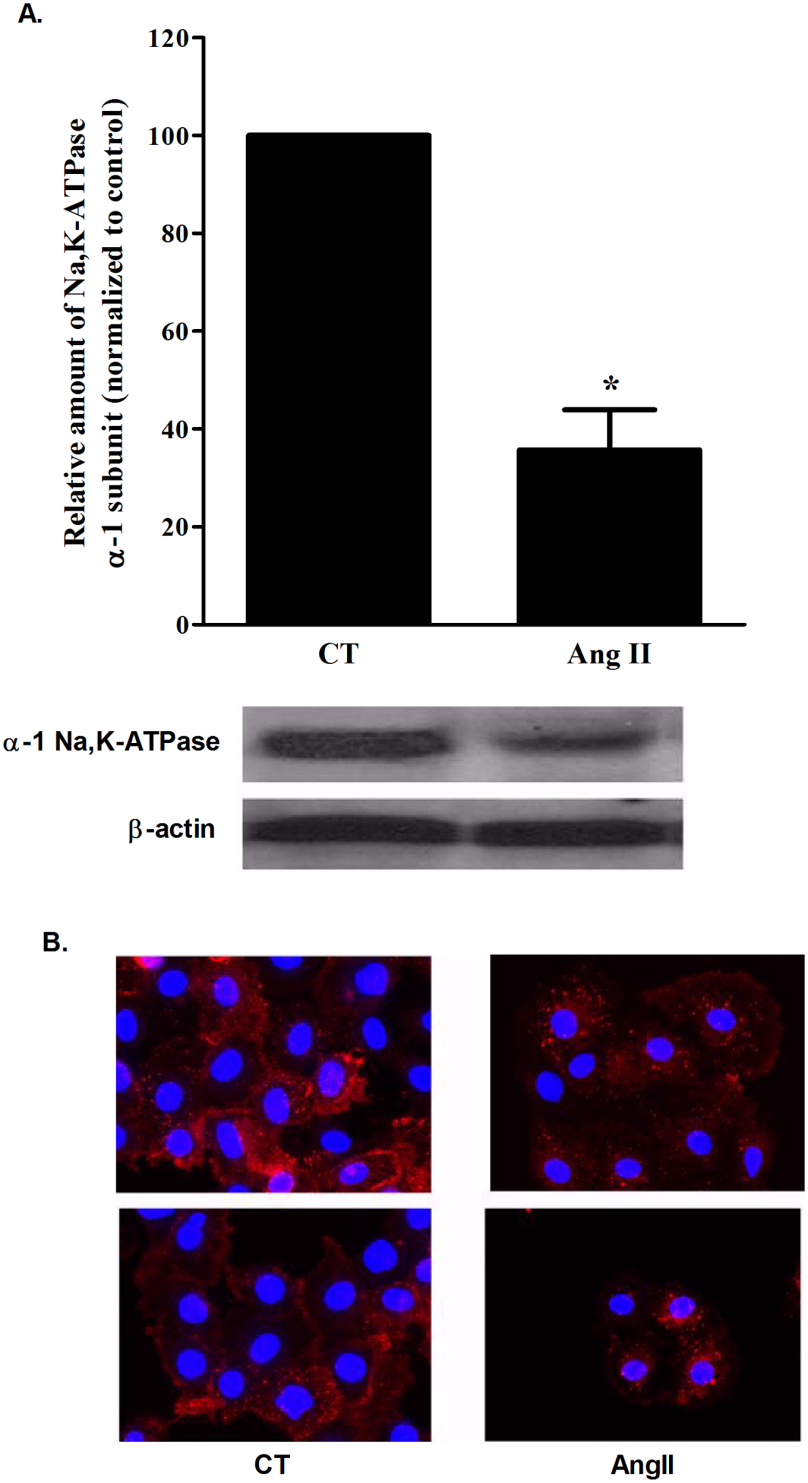
αNa,K-ATPase levels. (A) Na,K-ATPase α1 subunit levels in whole cell AEC II were measured by western blotting. Ang II administration decreased Na,K-ATPase levels by 0.65 folds compared to the control group. * P < 0.001 As compared to control group. CT—Control. Ang II—Angiotensin II. The bars represent mean ± SEM. (B) A representative Immunofluorescence staining of α1-Na,K-ATPase shows a shift of the protein membrane localization to internal organelles following Ang II administration. CT—Control. Ang II—Angiotensin II.

The levels of α-Na,K-ATPase were significantly decreased in Ang II (10^-7^M) treated cells when compared to untreated cells; from 100% in untreated cells to 45.8±18% in Ang II treated cells (P<0.001) (Fig 4A). Immunoflurescence staining showed a shift from a AEC II membrane to the inner organelles, suggesting an internalization of Na,K-ATPase (Fig 4B).

### cAMP involvement in AngII-induced reduction of Na,K-ATPase expression

**Fig 5:**
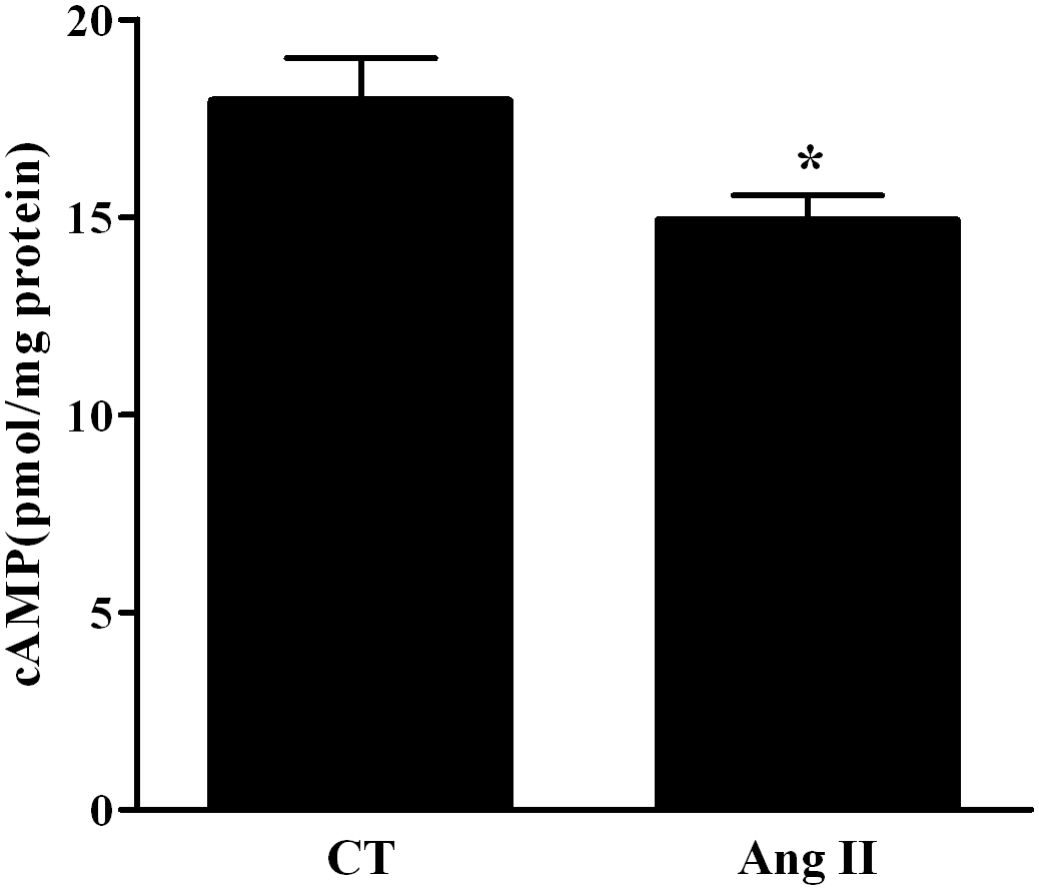
Levels of cAMP. Levels of cAMP were decreased in Ang II treated AEC II cells by 0.17 folds compared to the control group. * P < 0.05 As compared to control group. CT—Control. Ang II—Angiotensin II. The bars represent mean ± SEM.

It was previously reported that Na,K-ATPase can be regulated by cAMP pathway [15–17], thus we tested the theory of Ang II down regulating the effect on Na,K-ATPase through inhibiting the cAMP pathway. cAMP levels were measured using a cAMP immunoassay after 20 min of Ang II 10^−7^ M treatment. cAMP levels were significantly decreased in Ang II treated cells; from 17.74±2.4 pmol/mg protein to 14.9±1.4 pmol/mg protein, P< 0.05, (Fig 5 and S3 Table)

## Discussion

Renin-angiotensin-aldosterone system plays a vital role in body water balance. Angiotensin II regulates blood pressure and elicits dose-dependent decrease in renal blood flow and glomerular filtration rate [18]

The proteins repertoire of alveolar epithelial cells including Na,K-ATPase and ENaC that play a crucial role in active sodium transport and AFC and the recent evidence for existence of Ang II receptors in these cells [19]; led us to assume that Ang II may play a role in Alveolar fluid clearance with conceivable contribution to the pathogenesis of pulmonary edema.

Our results demonstrated that Ang II impaired AFC in a dose dependent manner (Fig 1). This may provide an additional mechanism to the development and resolution of pulmonary edema in CHF. Our results are consistent with the previously published data of Deng J. et al [20] who demonstrated the inhibitory effect of Ang II on AFC by using osmotic minipumps implanted in the internal jugular vein. Notably, in this report, the effect of Ang II on AFC might be due to systemic variables other than the lungs. Whereas, by using the isolated liquid filled-lungs model, we delivered angiotensin II directly to the lungs and thus examined solely the local effect of Ang II on AFC.

The isolated perfused liquid-filled lung has been extensively used to investigate active sodium transport and pulmonary edema clearance in the last two decades and proved to be reliable and simple. However, it has limitations. This model is physiologic, therefore, it is not possible to determine which cell type exactly contributes to the process of active transport and whether, it is solely via alveolar epithelium or both alveolar airway tissues. Rutschman et al stated that with consideration of the fact that the rate of active ion transport is low compared with passive bidirectional flux; very subtle variations may not be easily demonstrated (12). Although edema is simulated by filling with liquid and experiments are performed ex-vivo, this model has been shown to be suitable investigating the rate of pulmonary edema clearance.

In order to understand the underlying physiologic and molecular basis of the Ang II effect on AFC; we treated the lungs with Losartan, an angiotensin II receptor type 1 inhibitor. As shown in Fig 2A, Losartan restored the Ang II effect to control levels (from 5.2%±0.2 in Ang II treated lungs to 8.3%±0.2) (Fig 2A), suggesting that Ang II effect is mediated by AT_1_ receptor. Notably, the AT_1_ receptors were increased following the Ang II stimulation; this positive feedback intensifies the Angiotensin signaling cascade in AEC. AFC restoration by Losartan provides a new insight into Angiotensin derived pulmonary edema treatment options.

To evaluate whether the inhibitory effects of Ang II on AFC were contributed solely by AT1 receptor and in order to revoke AT_2_ role; we treated rats with the AT_2_ receptor antagonist (PD123319). Unexpectedly, PD123319 impaired AFC in control lungs (Fig 2A), suggesting that AT_2_ positively contributed to the substantial basal levels of AFC. Notably, the administration of both Ang II (10^-6^M) and PD123319, partially restored the rate of AFC as compared to Ang II alone. Based on these experiments, we presume first that AT_2_ basal triggering may be driven by low endogenous levels of Ang II. Second, the partial restoration of AFC may be due to the competitive binding of PD123319 and Ang II to AT_2_.

Furthermore, we reason that exogenous pharmacological Ang II levels mainly trigger AT_1_, thus decreasing AFC levels. Nevertheless, they also partly trigger AT_2_ by competing with PD123319 receptor binding, with consequent relative increase in AFC levels (S1 Fig). This hypothesis is strengthened by the shift of AFC levels between Ang II (10^-7^M) and Ang II (10^-6^M), yet it requires further investigation.

Downstream to AT_1_ receptor, cAMP pathway was diminished by Ang II treatment. It was previously reported that AFC proteins; including Na,K-ATPase and ENaC can be up regulated by the activation of cAMP pathway.

In order to investigate the contribution of Na,K-ATPase and ENaC to the AngII driven reduction of AFC; we treated rat lungs with Amiloride and Ouabain either alone or in the presence of Ang II. As shown in Fig 2A, the inhibitory effects of each, Ouabain and Amiloride, were augmented in Ang II treated lungs suggesting that both players; Na,K-ATPase and ENaC are under the effect of AngII stimulation.

Notably, our data is disconcordant with Ridge et al who observed that AFC was not decreased in rat lungs perfused with 10^−9^ M of Ouabain, These differences may stem from the route and duration of the drug administration. We have given Ouabain to the tail vein 30 minutes prior to scarifying the rat [21].

To further investigate AngII effect on Na,K-ATPase, its levels were measured in AngII treated/untreated AECII. α-Na,K-ATPase levels were decreased after AngII treatment; our results suggest that cAMP is involved in AngII-induced reduced Na,K-ATPase expression.,

ENaC has two different forms: the highly selective cation (HSC) channels which are the most numerous and highly selective for Na^+^, and the non-selective cation (NSC) channels with equal selectivity for Na^+^ and K^+^, HSC channels are typical ENaC made of the three subunits α, β and γ, whereas NSC channels are made of α-ENaC alone [22]. A preliminary experiment of measuring α-ENaC levels in AngII versus untreated AECII, did not show a significant change in α-ENaC (S2 Fig), while, at that time, Deng J. et al [20] demonstrated that Ang II has shifted the balance of these two forms in favor of the NSC, in rat lungs, by down regulating cAMP levels, thus reducing Na^+^ transport and conceivably down regulating water reabsorption.

**Fig 6:**
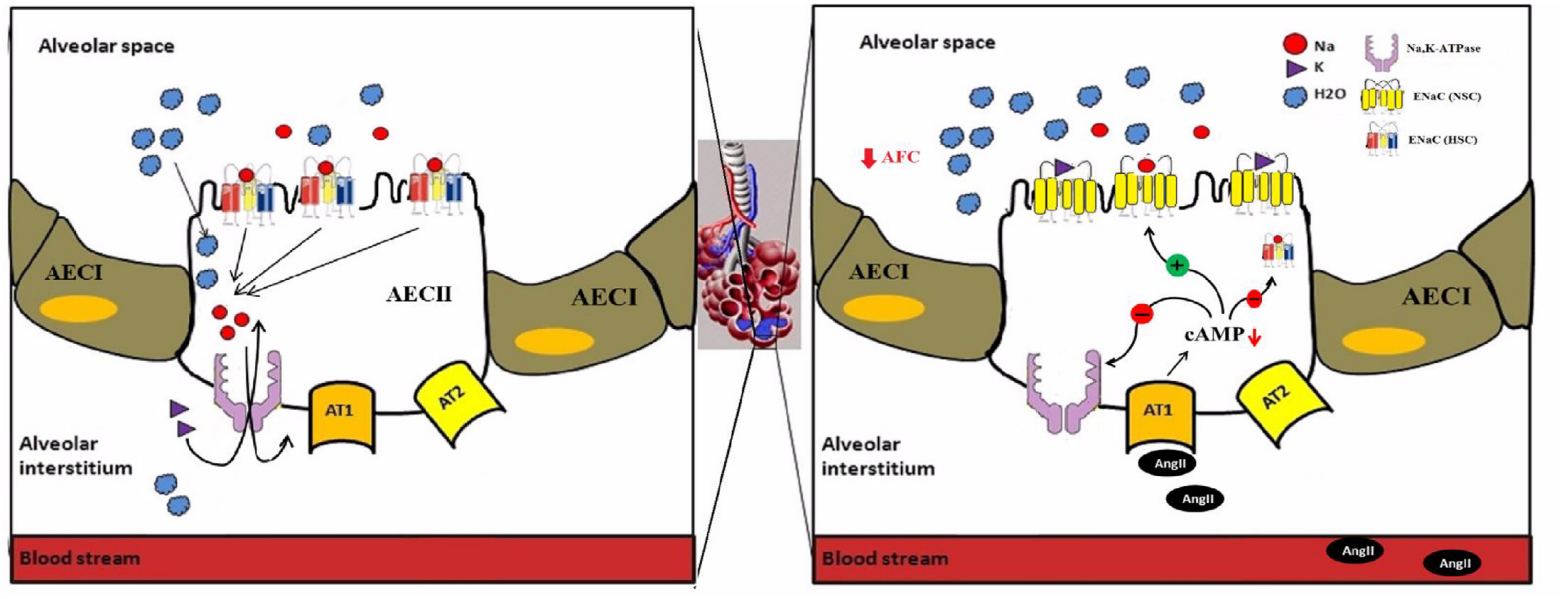
A comparable scheme of AFC under normal vs. Ang II stimulated conditions. Under normal conditions Na+ is extruded out of the alveolar airspace by apical epithelial Na+ channels (ENaC), specifically highly selective cation channels composed of α, β and γ subunits (HSC) and basolateral Na,K-ATPase pump with water following osmoticaly, Whereas Ang II stimulation down regulated cAMP levels in AEC II, via AT1 receptors triggering, thus leading to the decrease of the two important AFC players; αNa,K-ATPase and the HSC, and an increase of the NSC (non-selective cation channels composed of α subunit alone), with a resultant impairment of sodium reabsorption and conceivable AFC decrease.

These results alongside with our Na,K-ATPase findings provide a better understanding of Ang II inhibitory effect on alveolar active sodium transport and the deleterious effect on AFC. In summary, Ang II down regulated cAMP levels in AEC II, by AT_1_ triggering, thus leading to the decrease of the two important AFC players; α-Na,K-ATPase and the high selective channels for Na^+^; with resultant impairment of sodium reabsorption and conceivable AFC decrease (Fig 6).

This new information contributes to our understanding of the effects of Ang II in CHF. While its effect in the kidney is to promote salt and water retention; in the lungs, interestingly, it does the opposite and inhibits the process of active Na^+^ transport and water reabsorption.

Understanding the underlying mechanism of Ang II effect on AFC, is crucial for an adequate treatment, as it gives new perspective to the use of Ang II receptor blockers.

## Supporting Information

S1 Fig PD123319 competitive binding to AT_2_. A theoretical scheme describing the competitive interplay between AngII and PD123319 antagonist to AT_2_ receptor.
(TIF)

S2 Fig ENaC α subunit levels. α-ENaC levels in whole cell AEC II were measured by western blotting. Ang II administration did not show a significant change in αENaC levels compared to the control group. (105.4 ± 12.7% compared to 100%, respectively). # P > 0.05. CT—Control. Ang II—Angiotensin II. The bars represent mean ± SEM.
(TIF)

S1 Table Effect of Ang II on AFC. DOI: 10.6084/m9.figshare.1428529.
(XLSX)

S2 Table Different interventions effect on AFC. DOI: 10.6084/m9.figshare.1428530.
(XLSX)

S3 Table Levels of cAMP. DOI: 10.6084/m9.figshare.1428532.
(XLSX)
