# Correction: The Role of Angiotensin II and Cyclic AMP in Alveolar Active Sodium Transport

**DOI:** 10.1371/journal.pone.0137118

**Published:** 2015-08-26

**Authors:** Reem Ismael-Badarneh, Julia Guetta, Geula Klorin, Gidon Berger, Niroz Abu-saleh, Zaid Abassi, Zaher S. Azzam

The images for Figs 1 and 2 are incorrectly switched. The image that appears as Fig 1 should be Fig 2, and the image that appears as Fig 2 should be Fig 1. The figure captions appear in the correct order. Please see the correct Figs 1 and 2 here.

**Fig 1:**
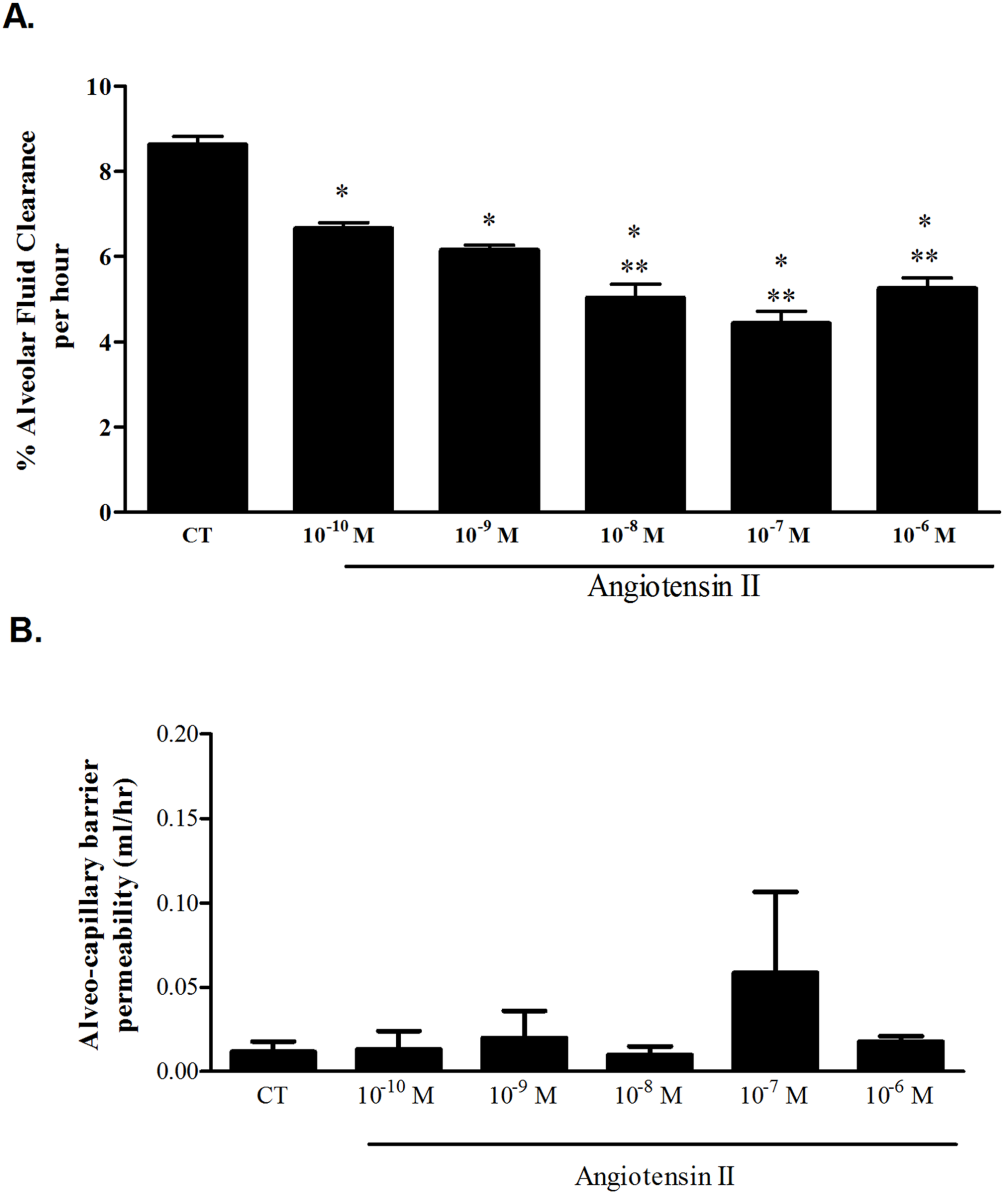
Effect of Ang II on AFC. (A) % Alveolar fluid clearance of the initial instilled volume was decreased in the Ang II groups in a dose dependent manner, from 8.6% ± 0.19 in control rats to 6.66% ± 0.13, 6.15% ± 0.11, 5.03% ± 0.31, 4.42% ± 0.29 and 5.25% ± 0.23 in Ang II (10^−10^ M, 10^−9^ M, 10^−8^ M, 10^−7^ M and 10^−6^ M) respectively. * P<0.001 As compared to control group; ** P<0.05 As compared to the rest of 10^−10^ M and 10^−9^ M Ang II treated groups. CT—Control. The bars represent mean ± SEM. (B) The albumin movement across the alveolar-capillary barrier did not differ significantly among the study groups indicating that the barrier was intact. CT—Control. The bars represent mean ± SEM.

**Fig 2:**
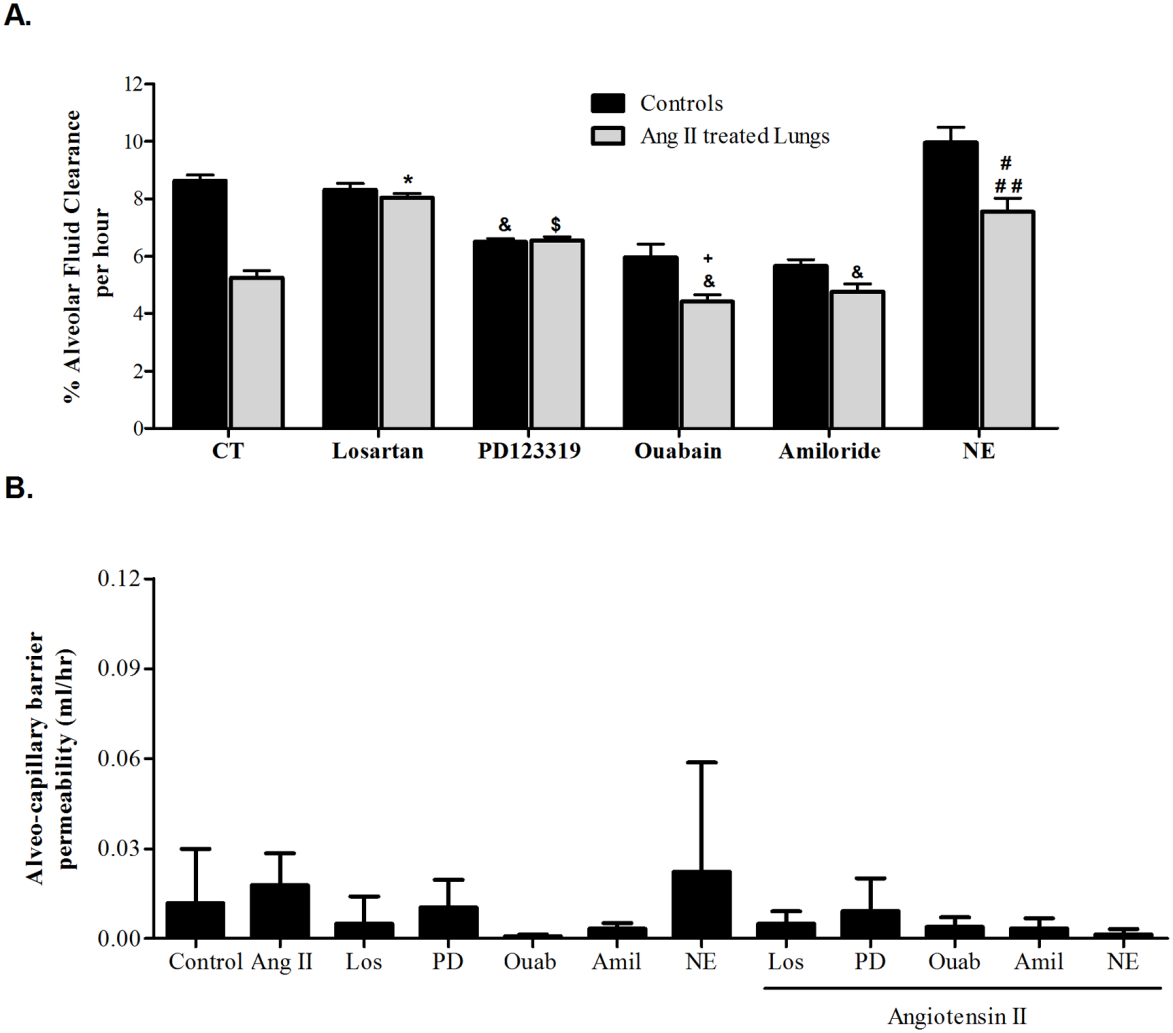
Different interventions effect on AFC. (A) Losartan restored Ang II effect on AFC from 5.25%±0.23 to 8.1%±0.13. AFC was not different in both losartan treated groups. * P<0.001 As compared to control group treated with Ang II. PD123319, AT_2_ receptor antagonist, decreased AFC in both AngII treated (n = 4) and untreated groups (n = 4) (6.54%±0.2 and 6.51%±0.2 respectively). $ P<0.05 as compared to Ang II group, & P<0.001 as compared to control group. Ouabain, the Na,K-ATPase blocker, significantly inhibited AFC in both control and Ang II treated rat lungs (5.9% ± 0.4 and 4.4% ± 0.2 respectively). + P<0.05 as compared to control rat lungs treated with ouabain alone. Amiloride, the sodium channel blocker, significantly reduced AFC in both control and Ang II treated rats as compared to untreated lungs (a 5.6% ± 0.2 and 5.01 ± 0.2 respectively). However, AFC was similar in the two Amiloride treated groups. Activating the adrenergic pathway by norepinephrine 10^-6^M increased the clearance percentage to 14.12% ± 1.8, when compared to control 8.6% ± 0.19. But when Ang II was also added, NE effect was abolished (7.3% ± 0.6). # P<0.05 as compared to control rat lungs treated with norepinephrine alone. ## P<0.0001 as compared to AngII group. CT—Control. Ang II—Angiotensin II. NE—Norepinephrine. The bars represent mean ± SEM. (B) The albumin movement across the alveolar-capillary barrier did not differ significantly among the study groups indicating that the barrier was intact. CT—Control. Ang II—Angiotensin II. Los—Losartan. PD—PD123319. Ouab—Ouabain. Amil—Amiloride. NE—Norepinephrine. The bars represent mean ± SEM.
