# Supplementary figures and images for: The Role of Angiotensin II and Cyclic AMP in Alveolar Active Sodium Transport
Source: PLoS One. 2015 Jul 31;10(7):e0134175. doi: 10.1371/journal.pone.0134175 (PMC4521808; doi:10.1371/journal.pone.0134175)

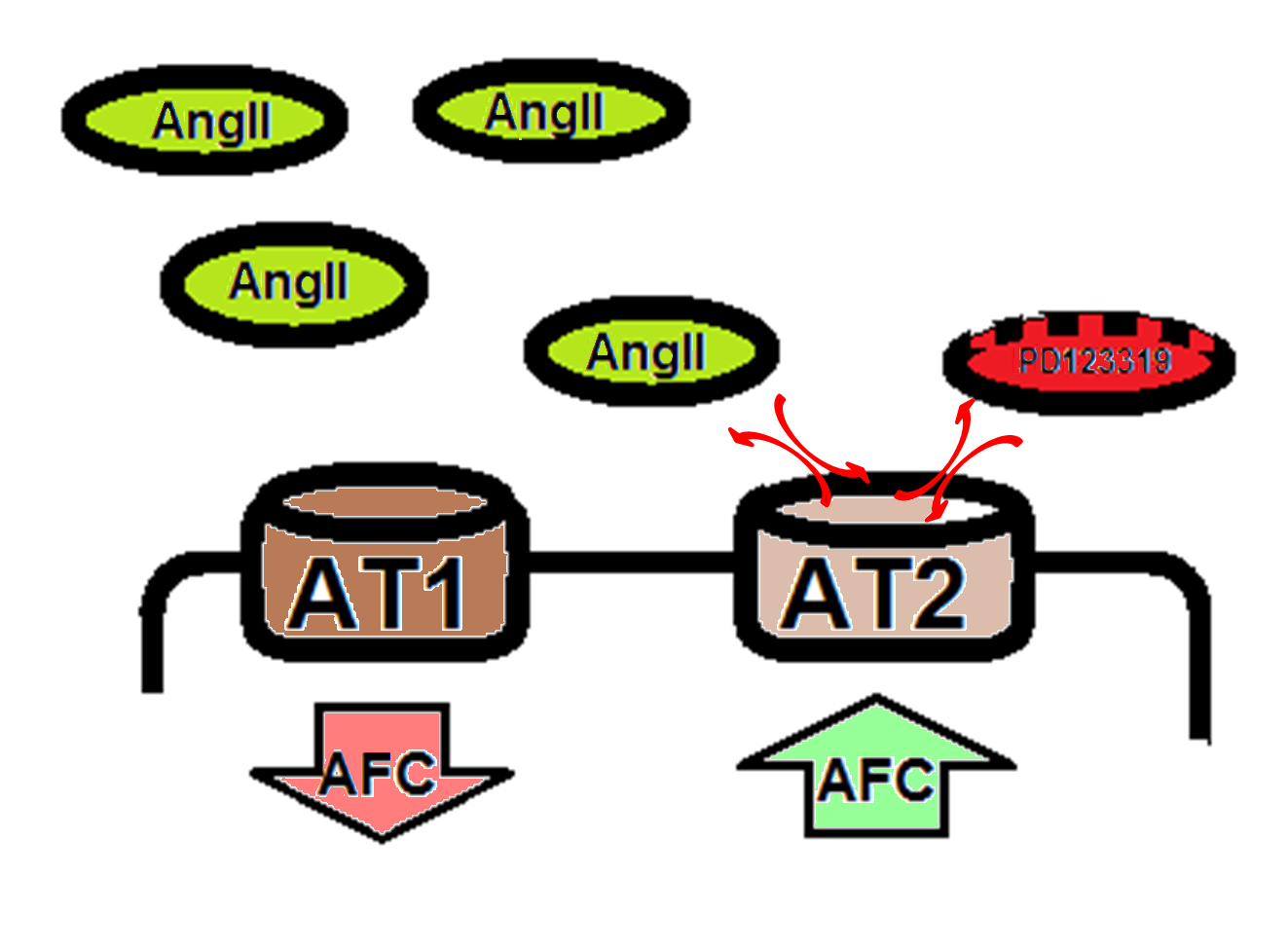

Supplement: S1 Fig — A theoretical scheme describing the competitive interplay between AngII and PD123319 antagonist to AT2 receptor. (TIF) [file pone.0134175.s001.tif]

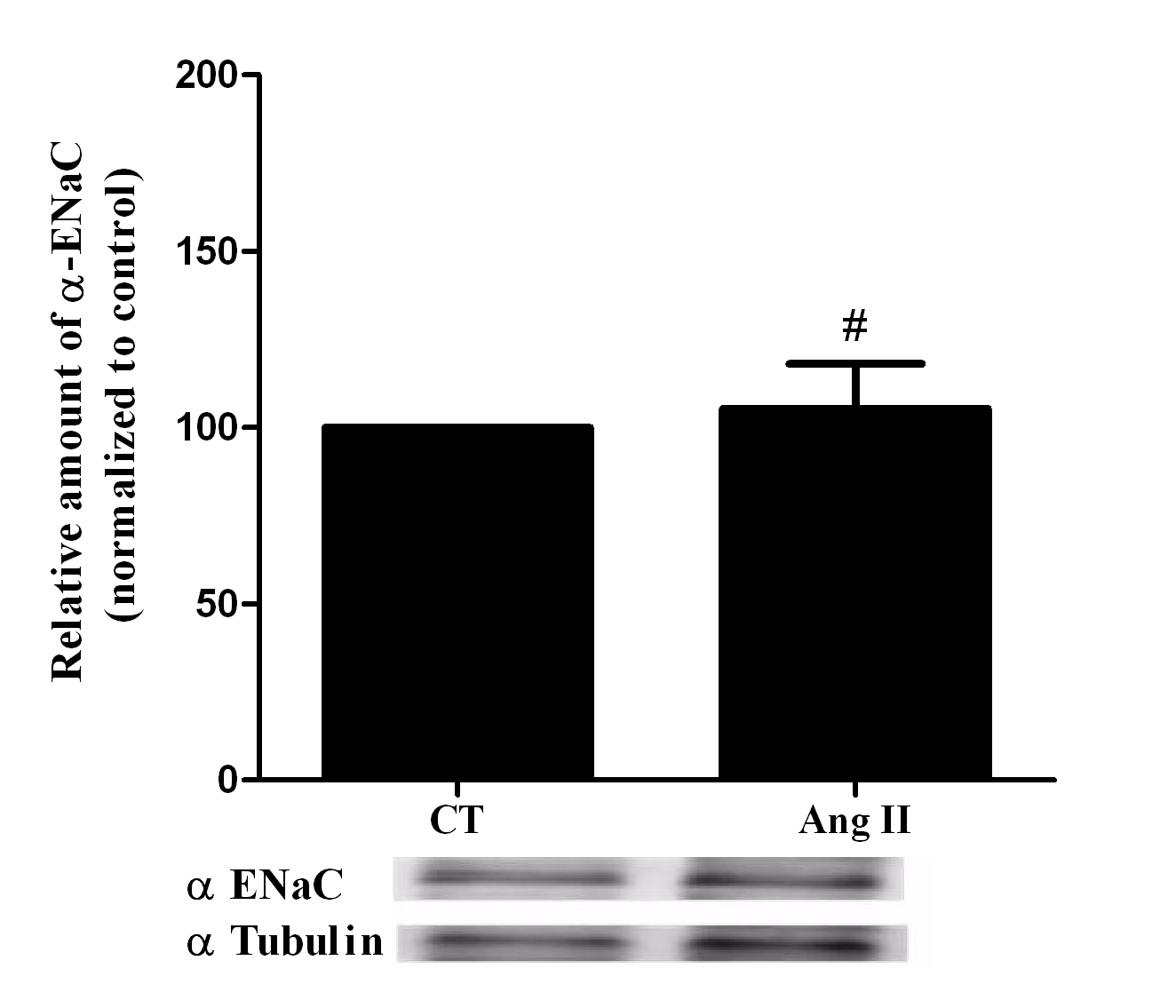

Supplement: S2 Fig — α-ENaC levels in whole cell AEC II were measured by western blotting. Ang II administration did not show a significant change in αENaC levels compared to the control group. (105.4 ± 12.7% compared to 100%, respectively). # P > 0.05. CT—Control. Ang II—Angiotensin II. The bars represent mean ± SEM. (TIF) [file pone.0134175.s002.tif]
